# Influences on influenza transmission within terminal based on hierarchical structure of personal contact network

**DOI:** 10.1186/s12889-015-1536-5

**Published:** 2015-03-18

**Authors:** Quan Shao, Meng Jia

**Affiliations:** College of Civil Aviation, Nanjing University of Aeronautics and Astronautics, 29 Jiangjun St., Nanjing, 211106 China

**Keywords:** Influenza spread, Passenger source partition, Immunity difference, Social relation structure, Personal contact network, Agent-based SEIR model

## Abstract

**Background:**

Since the outbreak of pandemics, influenza has caused extensive attention in the field of public health. It is actually hard to distinguish what is the most effective method to control the influenza transmission within airport terminal. The purpose of this study was to quantitatively evaluate the influences of passenger source, immunity difference and social relation structure on the influenza transmission in terminal.

**Methods:**

A method combining hierarchical structure of personal contact network with agent-based SEIR model was proposed to analyze the characteristics of influenza diffusion within terminal. Based on the spatial distance between individuals, the hierarchical structure of personal contact network was defined to construct a complex relationship of passengers in the real world. Moreover, the agent-based SEIR model was improved by considering the individual level of influenza spread characteristics. To evaluate the method, this process was fused in simulation based on the constructed personal contact network.

**Results:**

In the terminal we investigated, personal contact network was defined by following four layers: social relation structure, procedure partition, procedure area, and the whole terminal. With the growing of layer, the degree distribution curves move right. The value of degree distribution *p(k)* reached a peak at a specific value, and then back down. Besides, with the increase of layer *α*, the clustering coefficients presented a tendency to exponential decay. Based on the influenza transmission experiments, the main infected areas were concluded when considering different factors. Moreover, partition of passenger sources was found to impact a lot in departure, while social relation structure imposed a great influence in arrival. Besides, immunity difference exerted no obvious effect on the spread of influenza in the transmission process both in departure and arrival.

**Conclusions:**

The proposed method is efficient to reproduce the evolution process of influenza transmission, and exhibits various roles of each factor in different processes, also better reflects the effect of passenger topological character on influenza spread. It contributes to proposing effective influenza measures by airport relevant department and improving the efficiency and ability of epidemic prevention on the public health.

## Background

Since the earlier outbreaks of influenza A (H1N1) 2009 [1,2], influenza has grown to be a global contagious disease, and hundreds of thousands of people have died of influenza. Many scholars have tried to explore different ways to study influenza diffusion mechanism. For instance, agent-based model is one of these most important models. It is a micro-simulation model regarding individuals as agents or cells comprised of a cluster of behavior rules and finite states. Besides, the complex epidemic evolutionary behavior is consisted of etiology, host and environment. It is simulated by defining interactions of individual response and moving behavior in space in individual level [3-5]. As a result, spatial-temporal evolution process on epidemic transmission could get simulated effectively. Moreover, scholars have put forward a series of pedestrian flow models for the researches in crowd behavior and characteristic, such as cellular automata model, magnetic force model, queuing model, social force model [6], small world network [7-9] and so on. Nevertheless, these methods have inherent drawback in descripting heterogeneous individual, complex personal contact network among individuals and the autonomic behaviors.

In fact, the spread of influenza is interpersonal. To study the impact of individual contact behavior on influenza transmission, hierarchical structure of personal contact network is more suitable for the evaluation of spatial information in small space, such as geospatial location and spatial migration. The validity of the similar structures is verified with real data in some researches [10,11]. Therefore, we require a hierarchical spatial-topology structure to model personal contact network. It is feasible for analysis of influenza transmission in terminal.

On foundations above, we propose a method to quantitatively analyze the influences on influenza transmission in terminal by integrating agent-based SEIR model with hierarchical structure of personal contact network. The agent-based susceptible-exposed-infectious-removed (SEIR) model is used to describe dynamics of influenza transmission mechanism and passenger social relation structure is captured and quantified by utilization of hierarchical network of personal contact. We show that, with this method, it is possible to study the impact of passenger sources, immunity difference, and social relation structures on influenza diffusion. In addition, we also conclude the main infected areas during influenza spread. To evaluate the proposed method, a hierarchical personal contact network of a terminal is built based on passenger geospatial distribution. Transmission experiments of influenza are carried out in the generated network to simulate influenza evolution process. Besides, we analyze the impact of passenger topological character and spatial clustering of influenza spread. In consequence, it contributes to proposing effective influenza measures by airport relevant department and improving the efficiency and ability of epidemic prevention on the public health.

## Methods

### Probability distribution function of influenza transmission

As a respiratory infection, infectivity and rapid spread are the obvious features in influenza virus dissemination. Droplets in the air and contacts among individuals are considered as the major routes of influenza spread. The valid spread distance of droplets is usually less than 2 meters [12]. Moreover, the spread possibility has the relationship of valid contact time [13] and valid spread distance, it is also influenced by self-immunity.

The transmission probability density function *f*(*t*) varies with valid contact time *t*, has to satisfy Eq. (1) and Eq. (2). Thereby, Eq. (3) is deduced. Where, *β*_*i*_ represents infection rate of different immunity phase *i*.The probability distribution function *F*(*t*) is shown as Eq. (4).

1 $$ f(t)={\beta}_i\left(1-{\displaystyle \underset{0}{\overset{t}{\int }}}f\left(\tau \right)d\tau \right)\kern0.48em \left(0\le {\beta}_i\le 1\right) $$

2 $$ {\displaystyle {\int}_0^{+\infty }f(t)dt=1} $$

3 $$ f(t)={\beta}_i \exp \left(-{\beta}_it\right)\kern1.5em \left(0\le {\beta}_i\le 1\right) $$

4 $$ F(t)=1- \exp \left(-{\beta}_it\right)\kern1em \left(0\le {\beta}_i\le 1\right) $$

### Influential factors needs to be considered

#### Passenger sources partition

Population mobility is one of the important factors which push the fast influenza transmission. The floating population flows among different cities using vehicle as their carriers. Furthermore, air travel has greatly accelerated influenza diffusion and other diseases transmitted by person-to-person contact [14]. At the same time, terminal has a very mobile population, and gathers passengers from different regions. For this reason, we divide passenger sources into affected areas and unaffected areas. Passengers coming from affected area carry flu virus into local. Moreover, the airport services and procedures make passenger interval reducing, while the possibility of getting infected increasing. Therefore, flu virus would be extensively spread due to the daily population flows coming from affected areas. Based on these, the control of interactive behaviors among passengers from affected area and unaffected areas plays a critical role in influenza spread intervention.

#### Immunity difference

The spreading and infection of influenza will be affected by individual immunity, and the individuals with different immunity have their own resistance to the grippe. Hence, individual immunity difference should be taken into account while studying the influenza spread characteristics. Generally speaking, immunity difference is associated with the effects of ageing. As the increasing of age, individual immunity has showed the tendency to first increasing then decreasing during one’s lifetime. According to the survey of influenza spread characteristics and the estimation of influenza infection rate in Dr. Huang SQ’s doctoral dissertation in 2010 [15], we reset influences of influenza spread aimed at the population distribution characteristics and influenza spread process in airport terminal. Then, the influenza infection rates of people in different immunity phases are concluded based on influenza valid spread distance, contact distance, contact time, and self-immunity. Therefore, Table 1 lists the infection rate *β*_*i*_ (0 ≤ *β*_*i*_ ≤ 1) of passengers in different immunity phases when exposed to pathogens. Where, the highest infection rate lies in infants and juveniles, who have relatively low immunity of a group, and followed by elderly people, while the lowest infection rate appears to adults.

**Table 1:** **The infection rate of passengers in different immunity phases**

**Immunity phases**	**Infants and juveniles(<19)**	**Adults(20–60)**	**Elderly people(>60)**
Infection rate(*β*_*i*_)	*β*_1_ = 37.56%	*β*_2_ = 8.37%	*β*_3_ = 16.25%

#### Social relation structure

Social relation structure represents the frequent degrees of contact relation among passengers. It is impossible for one passenger to contact all passengers at the same time. Therefore, in order to identify the effective contact for influenza virus transmission, we classify social relation structures [16] of passengers into some categories based on the spatial distance between individuals [11], namely, relatives, colleagues, friends and other types of interpersonal relationship, which results in the spread of influenza in social network. Moreover, passengers in different social relation structures have diverse intimate relationships. Walking characteristics among passengers with different relationships are distinctly various, which lead to the difference in valid contact time *t* in Eq. (4). In the research of influenza spread, complex interpersonal relationship is extracted from hierarchical structure of personal contact network.

### Model modifications

#### Agent-based SEIR model

Agent is an individual with sociality, autonomy and activity. With the change of environment, they alter their behaviors and states according to changes in their environment [17]. To discuss influenza transmission characteristics in terminal, rules of agent-based SEIR model are proposed as follow:

1. There are four mutually exclusive disease states of agents facing flu during the diffusion, S (susceptible), E (exposed), I (infectious) and R (removed) [18].The state transition diagram can be shown as Figure 1.

**Figure 1:**
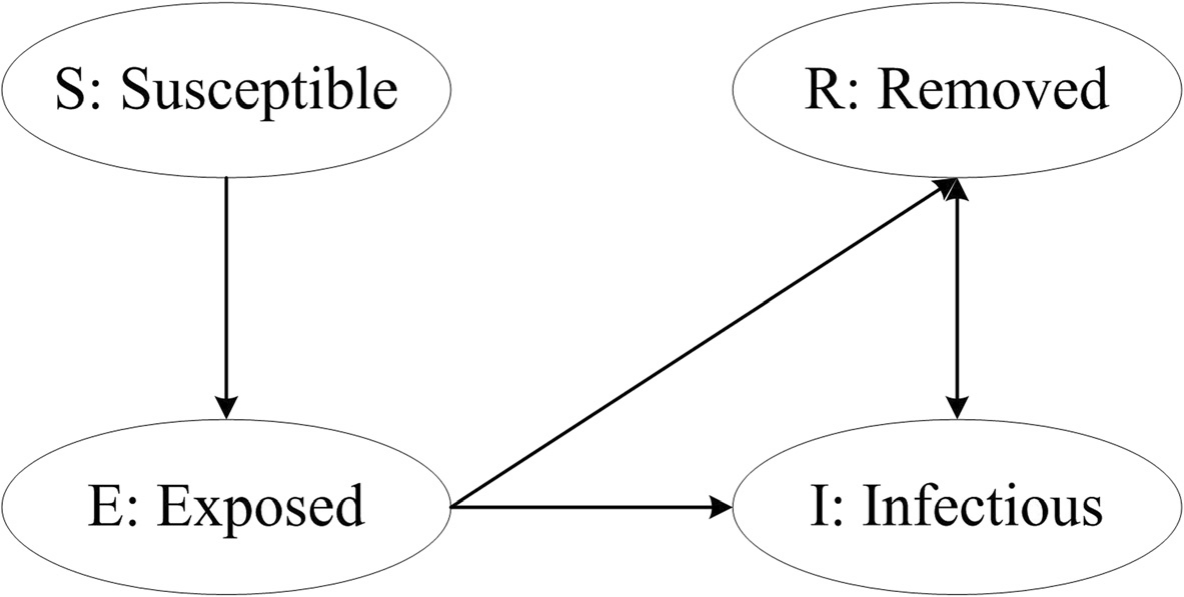
**The state transition diagram of individuals in four states.**
2. Susceptible agents could be converted into exposed state after contact with an infectious agent. Eq. (4) determined the functional relationship between infected probability *F*(*t*) and effective contact time *t* [19,20].
3. Exposed agents did not have infection ability. They could remain latent state or convert to infectious agent after contact with an infectious agent during the residence time in the terminal.
4. Infectious agents might infect agents into exposed state or infected state during their stay in terminal.
5. Removed agents had no ability to infect or be infected again because they were immune now.

#### Hierarchical structure of personal contact network model

The hierarchical structure of personal contact network is composed of multi-layer networks, and each layer contains several independent networks with same attributes are contained in each layer [11].

The first layer of hierarchical network (*α* = 0)is the unit model, which describes the most frequent contact relations, such as passengers in the same family and passengers in a travel group. Individuals in a unit model have a high likelihood to contact each other. We use node number *n* to control network topology. All the unit models in a whole network is a set {*n*_*i*_}. Each unit has a high clustering coefficient *C* = 1. The number of basic unit model *M* is:

5 $$ M=\frac{N}{n_0} $$

where *N* is number of nodes in a personal contact network. *n*_0_ is the number of nodes in all the unit models.

Based on the unit model, we can construct the hierarchical network of personal contact. It connects nodes from unit model to the whole hierarchal contact network. The unit models are called “sub-networks”, which are applied to build the higher layer networks. To generate the next layer of hierarchical network (*α* = 1) or a higher layer is a two-step process: firstly, the number of sub-networks is fixed. Secondly, new edges are added among these sub-networks to form a higher layer network. The newly constructed network *W* is as follow:

6 $$ \left\{\begin{array}{l}U={u}_1\cup {u}_2\cup \cdots \cup {u}_i\\ {}V={v}_1\cup {v}_2\cup \cdots \cup {v}_i+{v}_{new}\\ {}W=\left(U,V\right)\end{array}\right.,\kern1em i\in \left[1,{n}_x\right] $$

where *u*_*i*_ and *v*_*i*_ denote node set and edge set in a unit model *i* respectively. *v*_*new*_ represents the newly added edges. *n*_*x*_ is the number of unit models that compose the higher layer network. *U* and *V* mean all nodes and edges in network *W*.

Here, to fix the number of sub-networks, we use both spatial distance and social relation structure to estimate number of abstracted layers and number of sub-networks in a layer. Besides, we define a probability distribution function *F*(*y*) [11] to decide whether to add a new edge between individual *i* and individual *j* in different sub-networks.

7 $$ F(y)=\frac{e^{-\alpha}\left(\frac{d_i}{d_{i \max }}+\frac{d_j}{d_{j \max }}\right)}{2} $$

in which, *d*_*i*_ is the actual degree of individual *i* in layer *α. d*_*i* max_ expresses the maximum degree of links that individual *i* owns in layer *α* − 1.

The spread of influenza takes on the characteristics of hierarchical structure of personal contact network model. Moreover, passengers in different relationships have obvious different walking characteristics, among which the spatial distances *l* and intimate relationships *ω* are also various. Therefore, we propose a hierarchical structure of personal contact network model, in which passengers are regarded as nodes. These nodes are often provided with social relationships and geographic locations [21]. Personal contacts among passengers are regarded as network edge to connect nodes.

**Figure 2:**
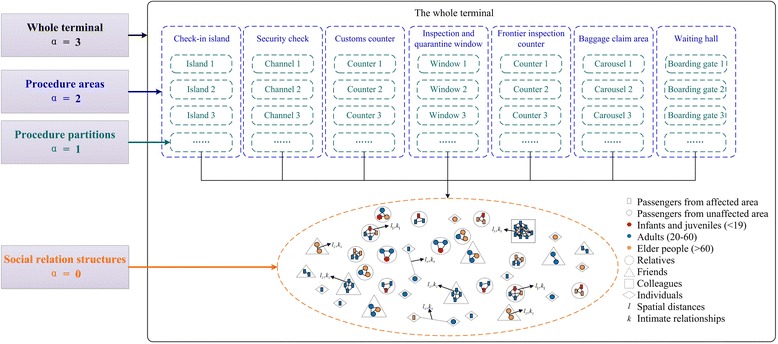
**The hierarchical structure of personal contact networks in terminal.**

In the investigated terminal, personal contact networks are defined by following four layers: social relation structure, procedure partition, procedure area, and the whole terminal, which is shown as Figure 2. Firstly, the contact network of same social relation structure is defined as the unit model *α* = 0. All the passengers are assigned a kind of social relations. The next layer is procedure partition contact network (*α* = 1). Contacts in this layer are slightly weaker than that in the same social relation structure. Each procedure partition is composed by four kinds of social relation structures, and new edges are added among passengers in the same procedure partition. The connection possibility is calculated according to Eq. (7). Then, procedure area (*α* = 2) and the whole terminal (*α* = 3) are generated in turn. Details will be shown in the experiments later.

In consequence, influenza spread model based on hierarchical structure of personal contact network is put forward to consider influenza transmission mechanism among passengers with different social relation structures, and it is also useful to predict the spread of epidemics.

## Results

### Experimental groups and initialization

To validate the model mentioned above, we apply this method to analyze the transmission process of influenza diffusion within a terminal. We carry out five sets of simulative experiments to study the impact of influenza diffusion factors. They are (1) considering passenger source partition only, (2) considering immunity difference only, (3) considering social relation structure only, (4) without considering the three factors mentioned above, and (5) taking account of passenger source partition, immunity difference and social relation structure at the same time.

According to the proportion of susceptible, exposures, infections and recoveries during the period of epidemic outbreak in the research of Liliana Perez and Suzana Dragicevic in 2009 [22], we set the initial percentages of passengers’ states based on influenza spread characteristic in airport terminal, which respectively are 74.2% (susceptible), 9.6% (exposed), 4.2% (infectious) and 12% (removed) both in arrival and departure. Then, influenza spread mechanism will be simulated according to the rules of agent-based SEIR model proposed in this paper. In each experiment, the total number of passengers in arrival and departure are roughly 7680, among which the number of domestic passengers is more than that of international passengers, approximately 4.3 times.

### Assumptions of influential factors

#### Passenger sources partition assumptions

Passengers coming from affected area are likely to carry flu virus, which will result in the spread of flu virus in terminal. Therefore, the initial number of affected area passengers has made a great impact on the evolution process of influenza transmission. To simulate this process, the passengers in affected area are imported at the beginning of experiments. Table 2 lists the number of passengers coming from affected area in departure and arrival.

**Table 2:** **The number of passengers coming from affected areas**

**Scene**	**Passenger source partition**	**Passenger number**
Departure	Affected areas	16
Arrival	Affected areas	16

#### Immunity difference assumptions

Recently, the percentages of China civil aviation passengers in different immunity phase are 15.6% (infants, juveniles), 60.7% (adults), and 23.7% (the old) [23]. Combining with the infection rate *β*_*i*_ (0 ≤ *β*_*i*_ ≤ 1)in Table 1, this can be designed to analyze the impacts of immunity difference on influenza spread within the terminal.

#### Social relation structures assumptions

History statistics shows that the proportions of passengers classified by social relation structure above are 31.6% (relatives), 26.4% (friends) 20.2% (colleagues), and 21.8% (individuals) respectively [23]. According to passenger psychological states and social relation structures, spatial distances *l* and intimate relationships *ω* among passengers of different social relation structures may change. Table 3 provides the assumptions about the four social relation structures of passengers.

**Table 3:** **The assumptions of social relation structure**

**Classification of passengers**	**Passenger number**	**Spatial distances** ***l*** **/m**	**Intimate relationships coefficient** ***ω***
Relatives	uniform (2,3)	0 ~ 0.55	2.6
Friends	uniform (2,5)	0.55 ~ 0.8	1.8
Colleagues	uniform (2,8)	0.8 ~ 1.2	1.5
Individuals	1	1.2 ~ 3.6	0

#### Hierarchical structure of personal contact network model in terminal

We have made a survey on the social relation pattern of passengers in a Chinese terminal to get their spatial distribution.

**Figure 3:**
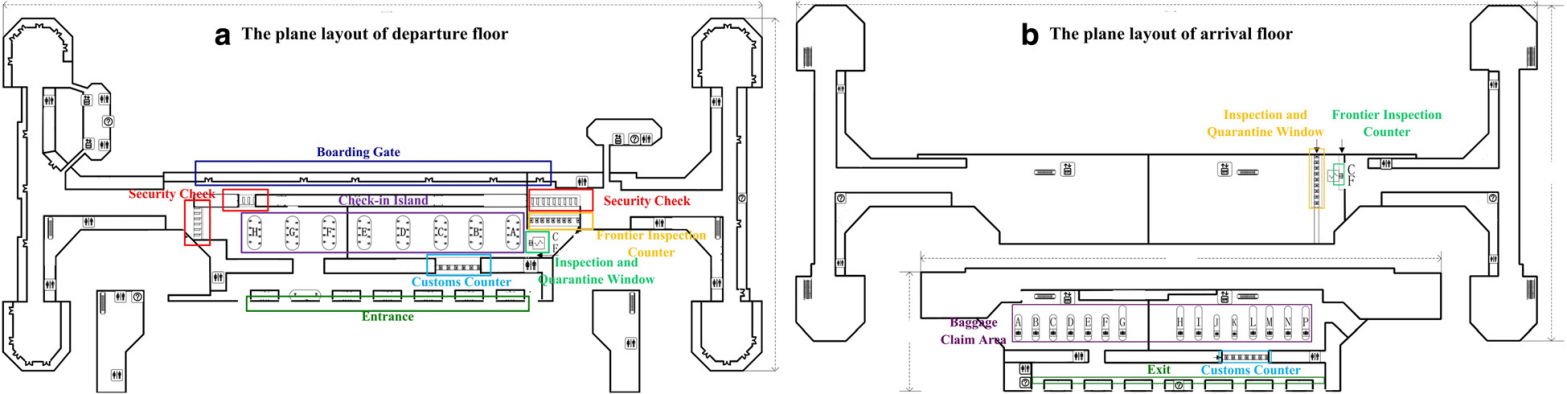
**The plane layout of terminal. (a)** The plane layout of departure floor (left). **(b)** The plane layout of arrival floor (right).

First, a research plan was set to make sure the purpose of survey. According to our research requirement, the investigation data included: the number of passengers in each social relation structure, the number of partitions in each passenger procedure, the number of procedure areas in each terminal, and passenger procedure distribution in the terminal. During the inspection in the terminal, we depicted the plane layout of terminal, which is shown as Figure 3. Meanwhile, we obtained passenger procedure distribution, the number of procedure areas and the number of partitions in each passenger procedure. There are seven procedure areas in this terminal. In addition, we counted the number of partitions in each passenger procedure, which respectively are 8 (check-in islands), 18 (security check channels), 6 (customs inspection counters), 2 (inspection and quarantine windows), 9 (immigration inspection counters), 15 (baggage carousels), and 30 (boarding gates).

Second, we obtained passenger social relation patterns by using the method of questionnaire investigation. A total of 400 questionnaires, included social relation pattern and number of passengers in each pattern, were issued to ticket sellers, check-in staffs and passengers in the terminal every day. After a 3-day investigation, 936 valid questionnaires were returned. According to the survey data, we summarized the major social relation patterns of passengers and calculated the average number of passengers in each social relation pattern, which respectively are 3 (relatives), 4 (friends), 6 (colleagues), 1 (individuals).

**Figure 4:**
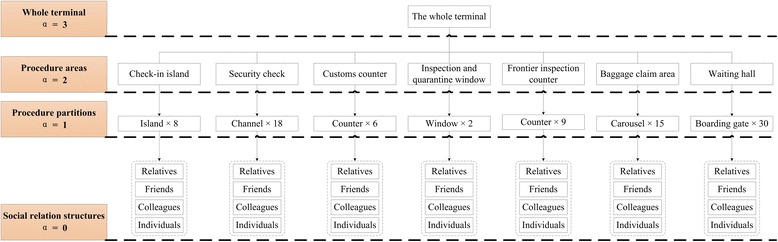
**The four layers of personal contact network in terminal.**

Based on the result above, the research data of passenger social relation patterns in the airport terminal are obtained. Moreover, we utilize these data to initialize the hierarchy of personal contact networks. The four layers of personal contact network are shown as Figure 4.

#### Terminal plane structure and passengers’ flow setting

The plane layout of a terminal is given as follow. Among them, Figure 3. (a) shows the plane layout of departure, where the entrance, check-in, security check, customs inspection, inspection and quarantine, immigration inspection, and boarding gate are offered. The left side of this floor is the activity area for domestic passengers, while the activity area for international passengers is in the right. Figure 3 (b) is the plane layout of arrival, in which the inspection and quarantine, immigration inspection, customs inspection, baggage claim, and exit are represented.

### The parameters of passengers’ flow

The experiment sets comfortable walking speed of passengers to be uniform distribution between 0.5 m/s and 1.0 m/s. Meanwhile, reference to domestic airport service commitment standard, time of passengers handing procedures is assumed as Table 4. Besides that, the arrival and departure time of passengers are determined in accordance with airport flight schedule. Passenger number is supposed randomly distributed between 100 and 120 on each flight.

**Table 4:** **Passenger scene setting**

**Procedures**	**Time (unit: min)**
Domestic check-in	Uniform(2,5)
International check-in	Uniform(3,8)
Security check	Uniform(3,10)
Immigration inspection	Uniform(1,2)
Customs inspection	Uniform(1,5)
Inspection and quarantine	Uniform(1,2)
Waiting for boarding	Uniform(15,90)
Baggage claim	Uniform(5,8)

## Discussion

In the each experiment, we adopt the method combing hierarchical structure of personal contact network model with agent-based SEIR model to randomly simulate influenza transmission in the terminal. Each experiment simulates 25 times.

### Hierarchical network properties

**Figure 5:**
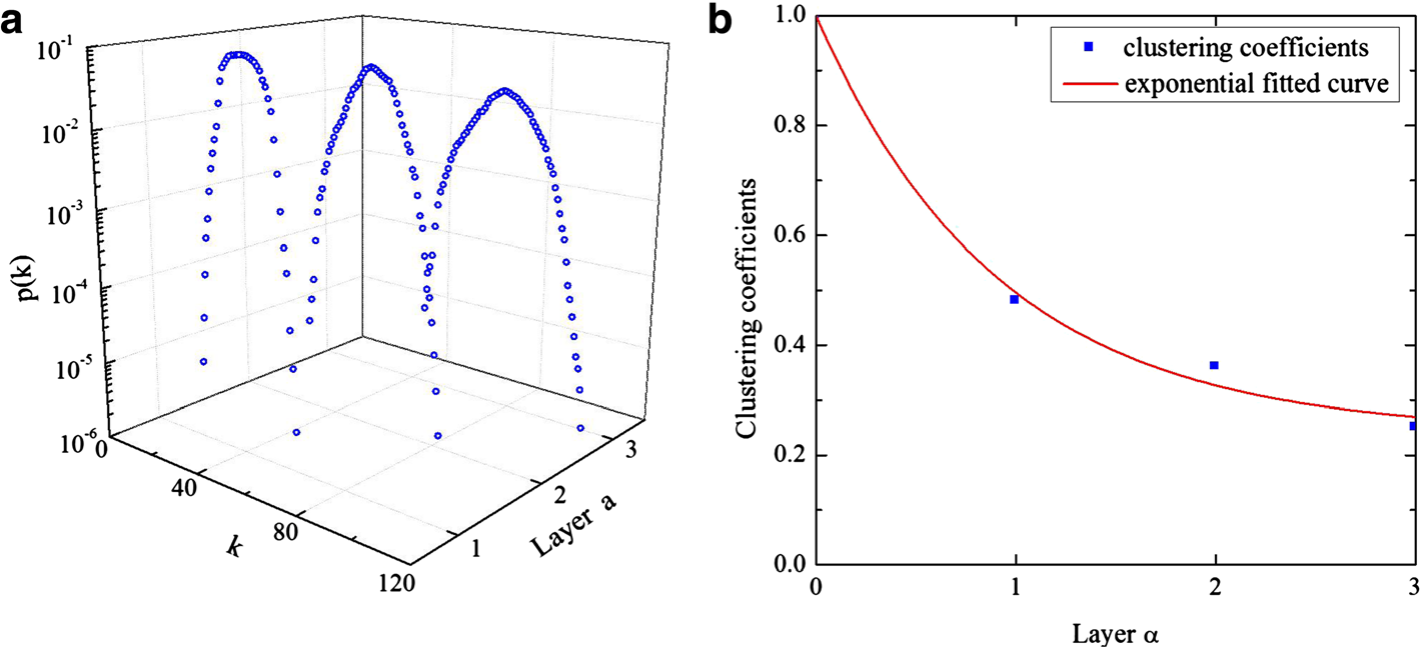
**The degree distributions and clustering coefficients of each layer personal contact network. (a)** The degree distributions of three layers personal contact network, *α* = 1, 2, 3 **(b)** The clustering coefficients of four layers personal contact network, *α* = 0, 1, 2, 3_._

From the social relation structure network (*α* = 0) to the whole terminal (*α* = 3), we calculate the degree distribution and clustering coefficients of generated personal contact networks for each layer, as shown in Figure 5. Figure 5 (a) shows the single-scale character [24] of degree distribution in each layer. With the growing of layer, the degree distribution curves move right. When degree *k* is small or large, degree distribution *p*(*k*) is approximately equal to 0. The value of *p*(*k*) reaches a peak at a specific value, and then back down. It means that passengers will contact more people when the spatial range expands. Besides, clustering coefficients presents a tendency to exponential decay with the increase of layer *α* in Figure 5 (b). It indicates that personal contact is frequent in small space and gets less intimate with the expansion of spatial range. As a consequence, the local clustering topology of personal contact network constrains spreading speed and scale of influenza. And influenza diffusion is limited by passenger contact patterns. This effectiveness is crucial for controlling influenza transmission and making interventions.

### Influences on influenza transmission

To identify the influence of passenger source, immunity difference and social relation structure on influenza transmission in terminal, we carry out influenza transmission experiments by utilization of proposed model with consideration of passenger source, immunity difference and social relation structure, respectively.

**Figure 6:**
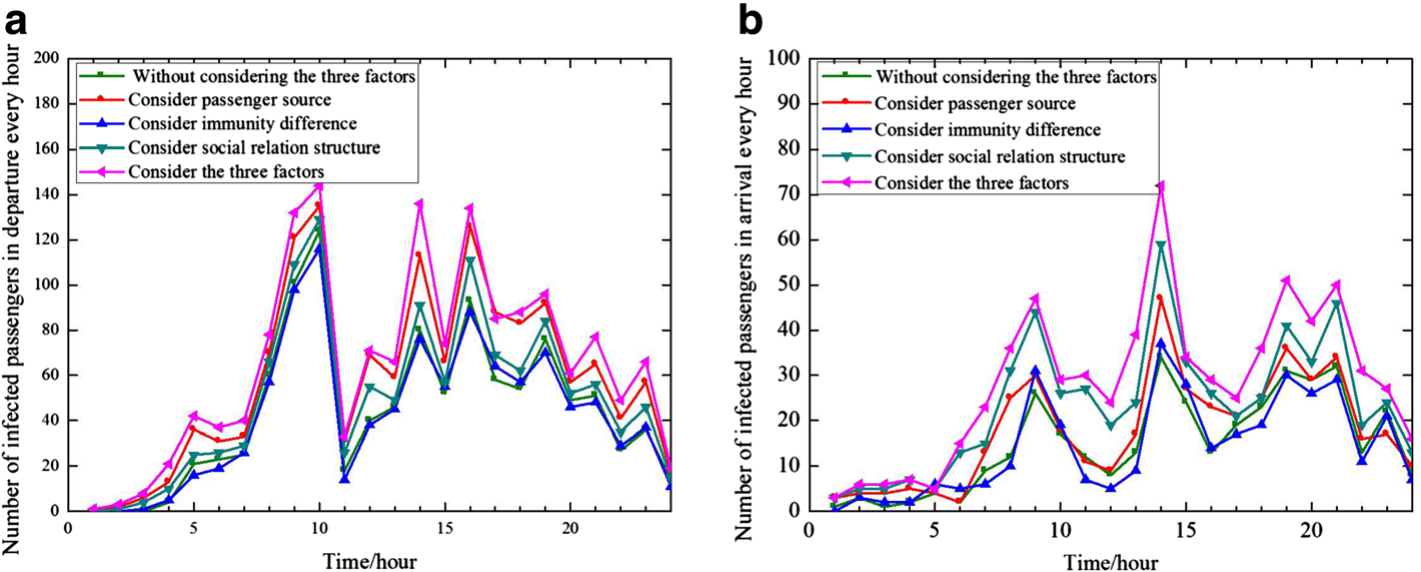
**The number of infected passengers in departure (left) and arrival (right) every hour.**

The average numbers of infectious passengers both in departure and arrival vary with time. They are respectively shown as Figure 6 (a) and Figure 6 (b). In the departure experiments, there are average 23.6 people infected when considering passengers coming from affected area, while 1.47 people contaminated by infectors without considering passenger source. Whereas, in the view of arrival experiment, 4.73 people get contaminated when passengers come from affected area and there are 0.26 people infected by infectious people ignoring the passenger sources. Furthermore, the total number of infectious passengers increases by 359 in departure all the day while it only grows by 72 in arrival in the consideration of passenger source partition. Hence, we argue that source of influenza virus carrier impacts a great on influenza spread in departure. Besides, passengers from affected area have the highest infectious ability when they handle domestic security, since there are 30.2 people effectively infected all the day.

The total number of infected passengers in departure and arrival are provided in Table 5. Contrast with the experiment irrespective of the three factors, the number of infectious passengers in departure experiment considering social relation structure grows by 155, while increases by 206 in arrival. And the experiment reveals that social relation structure plays an important role in arrival. Due to the different procedures in departure and arrival, passengers will get together to check-in or security check no matter what social relation structures they are in departure. Therefore, infectious passengers have more chances to contact and infect others. However, passengers of different social relation structures will separate after they disembark in arrival, which causes the reduction of the possibility of being infected.

**Table 5:** **The total number of infected passengers in departure and arrival**

	**Without considering the three factors**	**Considering passenger source partition**	**Considering immunity difference**	**Considering social relation structure**	**Considering all the three factors**
Departure	1052	1411	1016	1207	1561
Arrival	358	430	344	564	683

Compared with the number of infected passengers in experiment without considering the three factors, there exist no obvious changes in experiment considering immunity difference. So it may be concluded that immunity shows a little influence on influenza transmission in terminal. Probably because the immunity phase of China’s civil aviation passengers mainly assemble in adults which expresses nearly same infection rate.

In accordance with Figure 6, the peak period of infection appears mainly in 8:00 a.m.-10:00 a.m., 13:00 p.m.-14:00 p.m., and 15:00 p.m.-16:00 p.m. in departure, whereas in 8:00 a.m.-9:00 a.m., 13:00 p.m.-14:00 p.m., 18:00 p.m.-19:00 p.m., and 20:00 p.m.-21:00 p.m. in arrival. It indicates that spread ability of influenza is affected by passenger density. These periods possess the most flight movements, and more passengers handle procedures and wait for boarding in these periods, which result in smaller spatial distance among passengers.

### Main infected area

**Figure 7:**
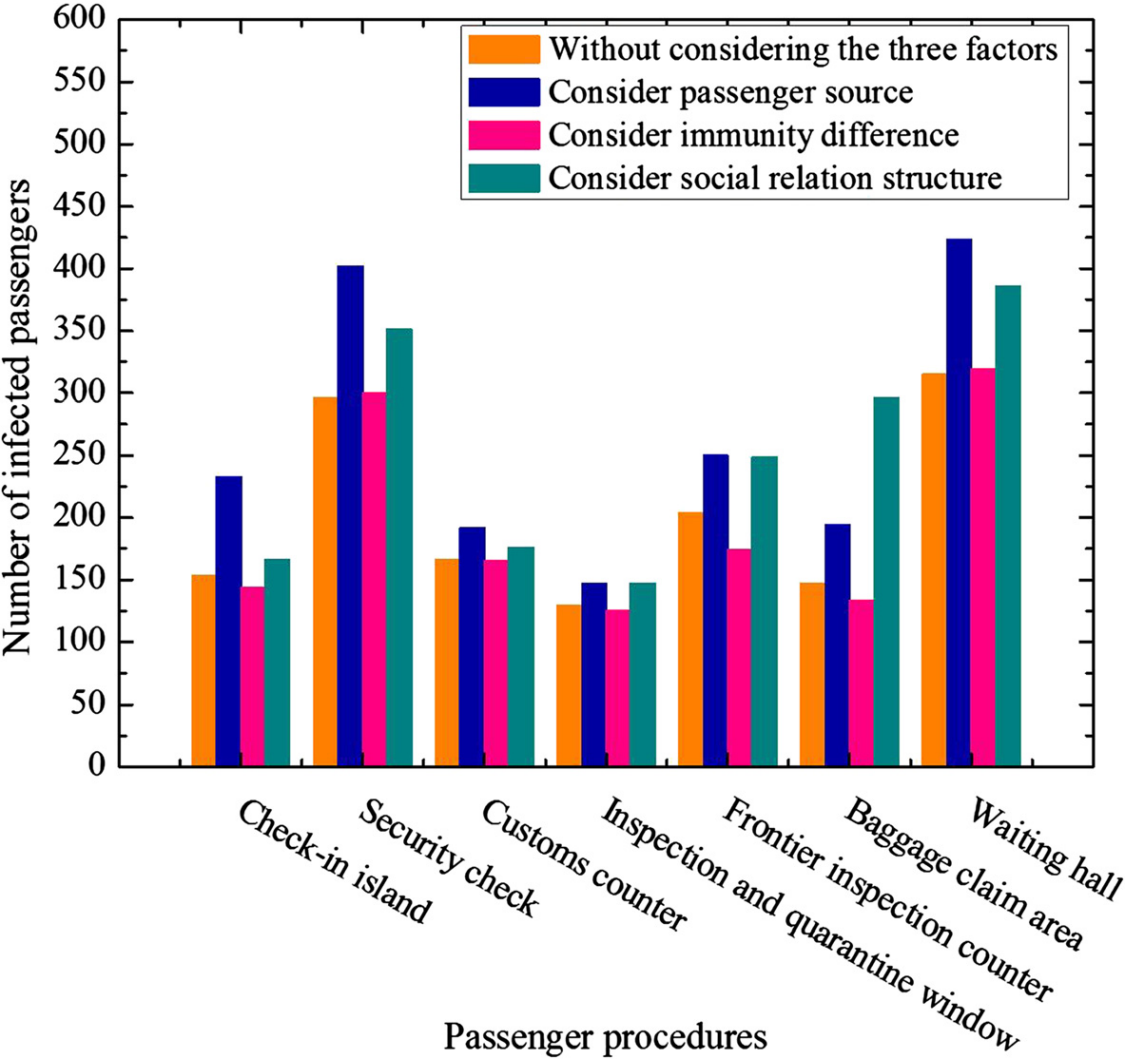
**The accumulative number of infected passengers in each procedure.**

In the light of Figure 7, the accumulative numbers of infected passengers in each procedure are not fully identical. From the view of Figure 7, we found that the number of infected passengers in security check and waiting hall is larger than that of other procedures. The main reason is that many passengers gather in security check and waiting hall, and the contact time is long. Thus, the longer contact time and smaller contact distance lead to these areas becoming the most likely place for influenza spread. In addition, compared with the experiment without considering the three factors, passenger source greatly influences the number of infected passengers in check-in, security check and waiting hall, which increase respectively by 80, 106 and 108. Accordingly, social relation structure plays a bigger role in baggage claim, and the number of infected passengers raise by 149. However, the influence of immunity difference in each procedure is not significant. Therefore, according to the different procedures in departure and arrival, we should focus on the key factors in each procedure and implement the most appropriate measures to handle with influenza transmission.

#### Intervention strategies

Based on the result discussed above, some efficacious intervention strategies need to be carried out to control the influenza prevalence, such as terminal disinfection, ventilation and reasonable partition. In addition, to shorten residence time, more service resources should be opened as earlier as possible, so as to reduce the valid contact probability. Moreover, the physical condition of passengers from affected areas would be regularly tested to stop those becoming infection sources in departure. Then, passengers with infected symptoms should be isolated immediately. Besides, endemic area isolation would be implemented in infectious groups in arrival. Passengers on affected flight are assigned to special gates, baggage carousels, immigration inspection counters, customs inspection counters, inspection and quarantine windows and exits.

## Conclusions

In this paper, we propose a novel approach to organically combine hierarchical structure of personal contact network model with agent-based SEIR model based on individuals’ spatial distribution information in terminal. It also provides an effective method to integrate personal contact structure with network topologies. By analyzing and simulating the different factors that affect flu virus spread, we reveal the characteristics and evolution process of influenza transmission. The factor of passenger sources get researched with the assignment of certain affected area passengers. And the behaviors of passengers with different social relation structures get micro-simulated with the utilization of hierarchical structure of personal contact network model, which clearly exhibits spatial clustering features of individual distributions. Moreover, the consideration of the influences from other pedestrians and environment improves the accuracy of critical parameters in agent-based SEIR.

(1) The proposed model owns the capability to reproduce the influenza diffusion process and analyze influences on concerned factors. Then, the effect of passenger topological character on influenza transmission is analyzed. This effectiveness is crucial for controlling influenza transmission and making interventions.

(2) Based on the experiments, the dynamic evolution of influenza transmission is simulated. In the spread process, we conclude the main infected areas when considering different factors. Moreover, partition of passenger sources is found having marked impact in departure, while social relation structure imposes a great influence in arrival. Besides, individual immunity difference exerts no obvious effect on the spread of influenza in the transmission process both in departure and arrival.

(3) Aiming at the factors of influenza spread, effective intervention measures are put forward to control influenza transmission. Moreover, in order to enhance the effect of implementation, the most appropriate measures directed against the key factors in each procedure should be paid attention to. It can be offered to researchers interested in epidemics, especially face-to-face contact diseases (Table 6).

**Table 6:** **Parameters explanation**

**Symbol**	**Name**	**Value**	**Source**
*f*(*t*)	Transmission probability density function	-	Calculated by Eq. (3)
*t*	Valid contact time	- /min	Social relation pattern
*β*_*i*_	Infection rate of different immunity phase	*β*_1_ = 37.56%, *β*_2_ = 8.37%, *β*_3_ = 16.25%	Dr. Huang SQ’s doctoral dissertation
*F*(*t*)	Probability distribution function	-	Calculated by Eq. (4)
*α*	Layer of hierarchical network	0, 1, 2, 3	Structure of personal contact network
*n*	Node number	-	Structure of personal contact network
{*n*_*i*_}	Set of all unit model	-	Structure of personal contact network
*C*	Clustering coefficient	0 ~ 1	Figure 5 (b)
*M*	Number of basic unit model	-	Calculated by Eq. (5)
*N*	Number of nodes in a personal contact network	-	Structure of personal contact network
*n*_0_	Number of nodes in all the unit models	-	Structure of personal contact network
*W*	Newly constructed network	-	Calculated by Eq. (6)
*U*	All nodes in network	-	Calculated by Eq. (6)
*V*	All edges in network	-	Calculated by Eq. (6)
*u*_*i*_	Node set in a unit model *i*	-	Structure of personal contact network
*v*_*i*_	Edge set in a unit model *i*	-	Structure of personal contact network
*v*_*new*_	Newly added edges	-	Structure of personal contact network
*n*_*x*_	Number of unit models that compose the higher layer network	-	Structure of personal contact network
*F*(*y*)	Probability distribution function of adding new edge	-	Calculated by Eq. (7)
*d*_*i*_	Actual degree of individual *i* in layer *α*	-	Characteristic of network and structure of personal contact network
*d*_*i* max_	Maximum degree of links that individual *i* owns in layer *α* − 1	-	Characteristic of network and structure of personal contact network
*l*	Spatial distances	0 ~ 0.55, 0.55 ~ 0.8, 0.8 ~ 1.2, 1.2 ~ 3.6	Social relation pattern
*ω*	Intimate relationships	2.6, 1.8, 1.5, 0	Social relation pattern
*p*(*k*)	Degree distribution	10^−6^ ~ 10^−1^	Figure 5 (a)
